# Continuous hydrogen regeneration through the oxygen vacancy control of metal oxides using microwave irradiation

**DOI:** 10.1039/c8ra08055k

**Published:** 2018-11-13

**Authors:** Keumyoung Seo, Sang-Mi Jeong, Taekyung Lim, Sanghyun Ju

**Affiliations:** Department of Physics, Kyonggi University Suwon Gyeonggi-Do 443-760 Republic of Korea shju@kgu.ac.kr

## Abstract

The amount of hydrogen gas generated from metal oxide materials, based on a thermochemical water-splitting method, gradually reduces as the surface of the metal oxide oxidizes during the hydrogen generation process. To regenerate hydrogen, the oxygen reduction process of a metal oxide at high temperatures (1000–2500 °C) is generally required. In this study, to overcome the problem of an energy efficiency imbalance, in which the required energy of the oxygen reduction process for hydrogen regeneration is higher than the generated hydrogen energy, we investigated the possibility of the oxygen reduction of a metal oxide with a low energy using microwave irradiation. For this purpose, a macroporous nickel-oxide structure was used as a metal oxide catalyst to generate hydrogen gas, and the oxidized surface of the macroporous nickel-oxide structure could be reduced by microwave irradiation. Through this oxidation reduction process, ∼750 μmol g^−1^ of hydrogen gas could be continuously regenerated. In this way, it is expected that oxygen-enriched metal oxide materials can be efficiently reduced by microwave irradiation, with a low power consumption of <∼4% compared to conventional high-temperature heat treatment, and thus can be used for efficient hydrogen generation and regeneration processes in the future.

## Abstract 2

Continuous hydrogen generation could be performed using microwave irradiation with polyvinylpyrrolidone for low energy reduction.

## Graphical abstract

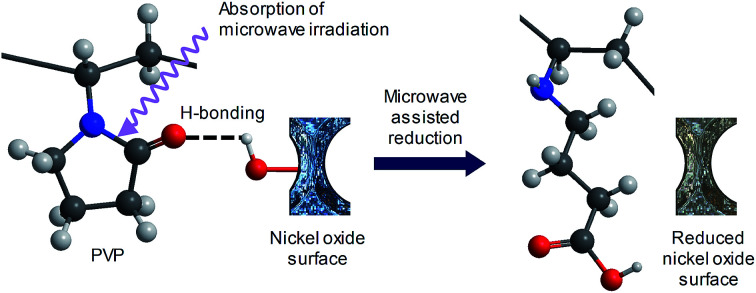

## 1. Introduction

Renewable energy is divided into new energy sources and renewable energy sources, which can replace existing energy sources, such as oil, nuclear, and natural gas. The representative renewable energies include solar, biomass, wind, small hydropower, geothermal and marine energy, and waste energy. The representative new energies include fuel cells, hydrogen energy, and coal liquefaction and gasification. Among them, hydrogen energy, called the clean energy of the future, belongs to the new energy field. Hydrogen energy is one of the ultimate alternative energy sources for the future. It has the advantages of water, which is a hydrogen-generating medium, existing abundantly on Earth and pollutants not being emitted during hydrogen combustion. It also has the advantage of eliminating problems such as noise when converting energy to a battery.

Hydrogen is produced from water, petroleum, coal, natural gas, and combustible waste. The conversion to hydrogen energy requires mediators such as electricity, heat, and microorganisms. Presently, steam reforming of petroleum or natural gas is a commercialized hydrogen generation method. However, there is the problem that when generating hydrogen using petroleum and natural gas, pollutants are generated, which is in conflict with the above-mentioned idea of hydrogen generation as clean energy. To overcome this issue, studies on hydrogen generation using electrolysis, thermochemical cycle, photocatalytic reaction, and biological decomposition methods are under way to obtain hydrogen from water.^1–4^ The thermochemical cycle is a method of generating hydrogen using the oxidation and reduction reactions of water at certain temperatures. Metal oxide materials are widely used for thermochemical hydrogen generation.^5–8^ Depending on the nature of the metal oxide material, the oxygen vacancies can fill in the metal oxide material at a relatively low temperature (800–1500 °C). However, high temperatures (1000–2500 °C) are required to create oxygen vacancies by spewing oxygen out of the metal oxide. Based on these properties, the reaction between H_2_O and the metal oxide material could induce water dissociation, where oxygen in the H_2_O fills the oxygen vacancies that exist in the metal oxide material and hydrogen is generated in the form of a gas, accordingly.

In currently-proposed hydrogen generation and regeneration technologies, there are still many problems in terms of energy efficiency because the energy required to generate and regenerate hydrogen is greater than or equal to the generated hydrogen energy. In order to solve these problems, studies are under way to develop a catalyst capable of generating and regenerating hydrogen using a low amount of energy. For instance, H_2_ generation and regeneration using Fe_3_O_4_/FeO, ZnO/Zn, In_2_O_3_/In_2_O, SnO_2_/SnO, CeO_2_/Ce_2_O_3_, and Mn_2_O_3_/MnO materials have recently been reported at the high temperature cycles of 800–1500 and 1000–2500 °C, respectively.^9–14^ In particular, research on lowering the temperature of hydrogen regeneration is essential for increasing the hydrogen generation energy efficiency. In recent years, efforts have been made to solve these problems. As examples of typical hydrogen regeneration studies using metal oxides, deep ultraviolet irradiation, water reduction catalysts, and solar heating methods have been introduced.^11,15,16^ However, it is still necessary to study novel hydrogen regeneration methods that can generate hydrogen repeatedly with a much lower energy.

In this study, we investigated the possibility of the continuous regeneration of hydrogen in a metal oxide catalyst using microwave irradiation. A macroporous nickel-oxide structure was used as the metal oxide catalyst for thermochemical hydrogen generation. We investigated how to reduce the macroporous nickel-oxide structure effectively and simply with a low energy compared to conventional hydrogen regeneration using external thermal energy. The efficiency of the reduction method using microwave irradiation was verified by comparing the amount of power that can regenerate hydrogen with the amount of power that regenerates hydrogen under high-temperature heat treatment. We also investigated how the polyvinylpyrrolidone (PVP) solution coated on the macroporous nickel-oxide structure promotes the oxygen reduction process during microwave irradiation. The surface characteristics of the macroporous nickel-oxide structure were analyzed by optical, physical, and chemical methods during the repetitive hydrogen regeneration process using microwave irradiation.

## 2. Methods

### 2.1 Hydrogen generation

The macroporous nickel-oxide structure (99.9% purity) with an internal pore size of 230 μm, an external size of 2.5 × 2.5 cm^2^, and a thickness of 0.5 mm to be used as a metal oxide catalyst for hydrogen generation was dipped in acetone for 10 min to remove organic/inorganic contaminants and was rinsed with deionized water for 5 min by the ultrasonic cleaning method. To completely remove the moisture remaining on the surface of the macroporous nickel-oxide structure, a thermal annealing process was carried out in a heat oven at 90 °C for 2 h. The ten prepared macroporous nickel-oxide structures were mounted on an alumina jig for hydrogen generation. He gas, which does not react with the macroporous nickel-oxide structure, was injected into the reaction chamber at a flow rate of 50 sccm. When the temperature reached 800 °C, a mixture of He gas/H_2_O vapor (200/100 sccm, respectively) was injected into the reaction chamber. It is worth noting that the H_2_O steam component, which was not decomposed by hydrogen, was separated into a cold trap. Under these conditions, the amount of hydrogen generated per minute in the macroporous nickel-oxide structures was measured by gas chromatography (GC, Agilent 7890B). The generated hydrogen flowed into the gas chromatograph at a volume of 4 cm^3^ every 4.3 min. The measured amount of generated hydrogen (cm^3^ min^−1^) was used to calculate the rate of hydrogen generation (mol min^−1^ g^−1^) using the equation *V* = *nRTP*^−1^ (*n*, number of moles of gas; *V*, volume; *R*, ideal gas constant; *T*, absolute temperature; *P*, pressure) and the mass of the loaded nickel-oxide structures. Correction fitting curves for accurate hydrogen generation were obtained using three He–H_2_ mixed standard gases with hydrogen concentrations of 0.02%, 1.03%, and 3.12%.

### 2.2 Hydrogen regeneration

After hydrogen generation, the ten oxidized macroporous nickel-oxide structures were dipped into a solution of PVP (Sigma-Aldrich, molecular weight ∼55 000) and diethylene glycol (DEG, Junsei, 99%) with a ratio of 1 : 5 as a reducing agent. The PVP-coated macroporous nickel-oxide structures were then placed in a household microwave oven capable of controlling the level of the output power. After the PVP-coated macroporous nickel-oxide structures were treated for 180 s at various microwave power levels ranging from 200–1000 W in 200 W steps, the coated PVP was removed by an NMP (*N*-methyl-2-pyrrolidone, Sigma-Aldrich) solvent. It was then cleaned in a DI water and isopropyl alcohol cleaning process (sonication for 10 min) and dried in a heat oven for 2 h. During the hydrogen generation and regeneration cycles, the surface morphology of the macroporous nickel-oxide structures was measured by a field-emission scanning electron microscope (FE-SEM) and the surface components were analyzed by X-ray diffraction (XRD). The pore states of the macroporous nickel-oxide structures before and after hydrogen generation/regeneration were analyzed by Brunauer–Emmett–Teller (BET) analysis. The oxidation states of the macroporous nickel-oxide structures during the reaction were characterized by X-ray photoelectron spectroscopy (XPS).

## 3. Results and discussion

Microwave irradiation in a microwave oven, which is widely used for cooking food in everyday life, has a long wavelength of 0.01–1 m, between infrared radiation and radio-waves in the electromagnetic spectrum. In order to differentiate between communication and microwave radar equipment, household and industrial microwave ovens operate at a fixed frequency of 2.45 GHz. The energy of a microwave photon is calculated to be 0.037 kcal mol^−1^ using Planck’s law (*E* = *hν*). Using a microwave, uniform and rapid heating can be achieved by inducing vibrational and rotational motion to the organic molecules that have a dipole moment. Particularly in the case of molecules that bond with a large dipole moment, such as C–O or C–N bonding, the vibrational energy of the bonding can easily absorb microwave energy and can quickly convert it to thermal energy. Although the energy in a microwave photon (0.037 kcal mol^−1^) is much lower than the typical energy required for breaking a molecular bond (80–120 kcal mol^−1^), the kinetic energy, which is accumulated by the irregular rotation and vibration of chemical bonding, can cause dipole bonding to become unstable, and enable a chemical reaction with the surrounding molecules.^17^ The efficient microwave-assisted reduction process, that can be used for the water splitting process using low energy microwave irradiation instead of conventional high energy heating, was proposed.

In the case of the two-step water splitting process using a metal oxide as a redox pair, the surface of the metal oxide material is oxidized through the oxidation step (MO_red_ + H_2_O (g) → MO_ox_ + H_2_) during the process of hydrogen generation, and a highly endothermic reduction step (MO_ox_ + external energy → MO_red_ + 1/2O_2_) is required for hydrogen regeneration.^1^ In this study, the PVP solution, which shows a good adsorption ability on the metal oxide surface by forming hydrogen bonds between the metal oxide surface and the polymer segment, was coated on the surface of the oxygen-rich macroporous nickel-oxide structure.^18^ We then developed the microwave irradiation method for the oxygen-rich macroporous nickel-oxide structures to be effectively reduced by the low microwave energy in a short reaction time.

**Fig. 1:**
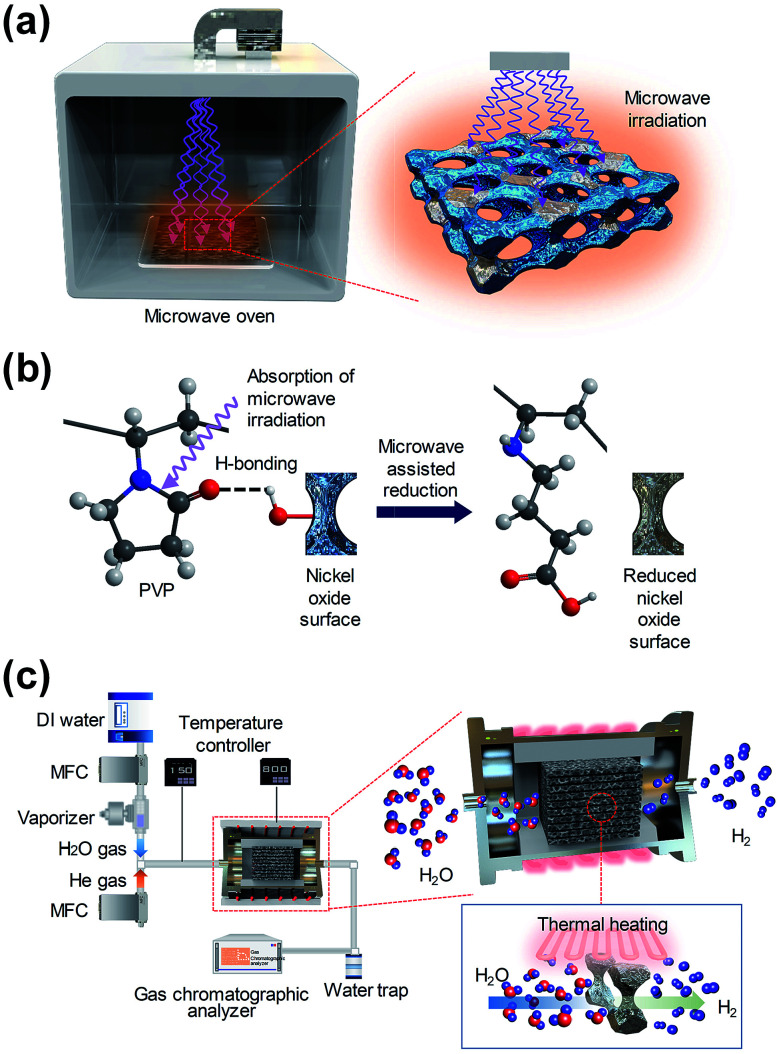
(a) Diagram of the macroporous nickel-oxide structure under microwave irradiation in a chamber. (b) Hydrogen regeneration process of the macroporous nickel-oxide structure for oxygen reduction under microwave irradiation with PVP. Nickel-oxide is reduced by the chemical reaction between the unstable heterocyclic moieties of PVP and the oxygen-rich nickel oxide surface caused by microwave irradiation. (c) Experimental setup for hydrogen generation.

Fig. 1(a) shows a diagram of the reduction step, which is followed by microwave irradiation on an oxygen-rich macroporous nickel-oxide structure after hydrogen generation to form an oxygen-vacancy-rich macroporous nickel-oxide structure. As shown in Fig. 1(b), during microwave irradiation, the macroporous nickel-oxide structure coated with PVP absorbs microwaves well due to the large dipole moment of PVP with a heterocyclic moiety. In particular, the absorbed microwaves induce the vibration and rotation of the C–N and C=O bonds constituting the heterocyclic moiety of PVP, and make the C–N and C=O bonds unstable, thus the metal oxide can be reduced by a chemical reaction between PVP and the oxygen which existed in the metal oxide that became hydrogen bonded.^17^ During the process, the oxygen located on the oxygen-rich macroporous nickel-oxide structure could be rapidly removed by microwave-assisted reduction. Through these procedures, it was possible to regenerate a similar amount of hydrogen gas repeatedly from multiple redox reactions. Fig. 1(c) shows a schematic of the hydrogen generation system using the macroporous nickel-oxide structure as a catalyst. Water vapor was passed through the ten square-shaped macroporous nickel-oxide structures with an internal pore size of 230 μm mounted on an alumina jig in the reaction chamber.

**Fig. 2:**
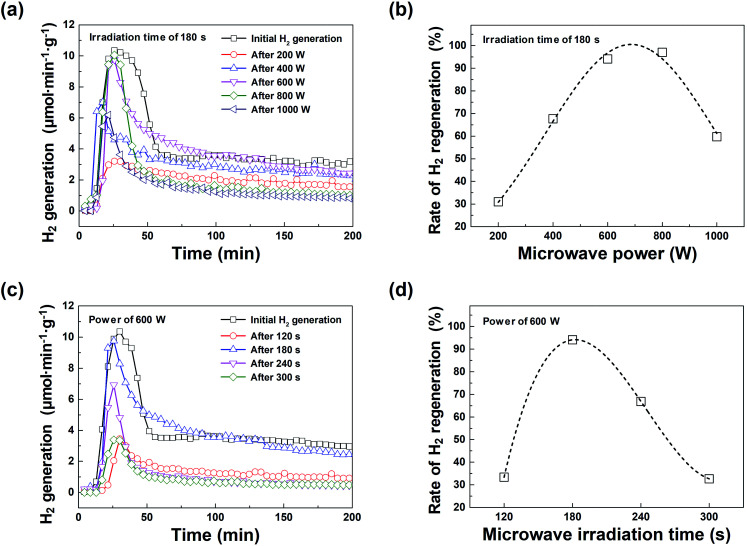
(a) Hydrogen regeneration by the macroporous nickel-oxide structure with varying microwave power. (b) Rate of hydrogen regeneration with varying microwave power. (c) Hydrogen regeneration by the macroporous nickel-oxide structure with varying microwave reduction time. (d) Rate of hydrogen regeneration with varying microwave irradiation time.

Fig. 2(a) shows a comparison of how the hydrogen regeneration amount differs from the initial hydrogen generation amount according to the microwave irradiation power conditions (200–1000 W in 200 W steps). As the microwave power increases, the amount of hydrogen regeneration also increases. At a microwave power of 600 W, the maximum hydrogen generation rate and the total amount of generated hydrogen were ∼9.8 μmol min^−1^ g^−1^ and ∼750 μmol g^−1^, respectively, which were similar to those of the initial hydrogen generation. It is worth noting that the temperature required for hydrogen generation was 800 °C. The maximum rate of hydrogen evolution was plotted as a function of the temperature to address the hydrogen generation effect of the macroporous nickel-oxide structure on the water-splitting reaction. The maximum hydrogen generation rate and the amount of hydrogen generated from the macroporous nickel-oxide structure, with the reduction process of microwave irradiation, were similar or slightly lower than the hydrogen generation values of conventional metal oxide materials, such as CeO_2_, Mn_3_O_4_, and FeAl_2_O_4_ (∼8.4–33 μmol min^−1^ g^−1^ at 850–1600 °C).^1,14,19,20^ The use of microwave irradiation can be regarded as a very efficient method to regenerate hydrogen because it does not require high-energy heat sources. As shown in Fig. 2(b), the hydrogen regeneration rate increases as the microwave power increases, and the hydrogen regeneration rate is at a maximum between 600 and 800 W. However, the amount of hydrogen regeneration decreases due to mechanical damage, notably surface loss and burning, of the surface of the macroporous nickel-oxide structure at microwave powers above 800 W. Fig. 2(c) and (d) show the hydrogen generation characteristics with varying microwave irradiation times of 120, 180, 240, and 300 s while the microwave irradiation power was fixed at 600 W. The hydrogen regeneration rate increased to 33.3% (120 s) and 94.1% (180 s), but reduced to 66.8% (240 s) and 32.6% (300 s) due to the burning phenomenon that occurred in the macroporous nickel-oxide structure.

**Fig. 3:**
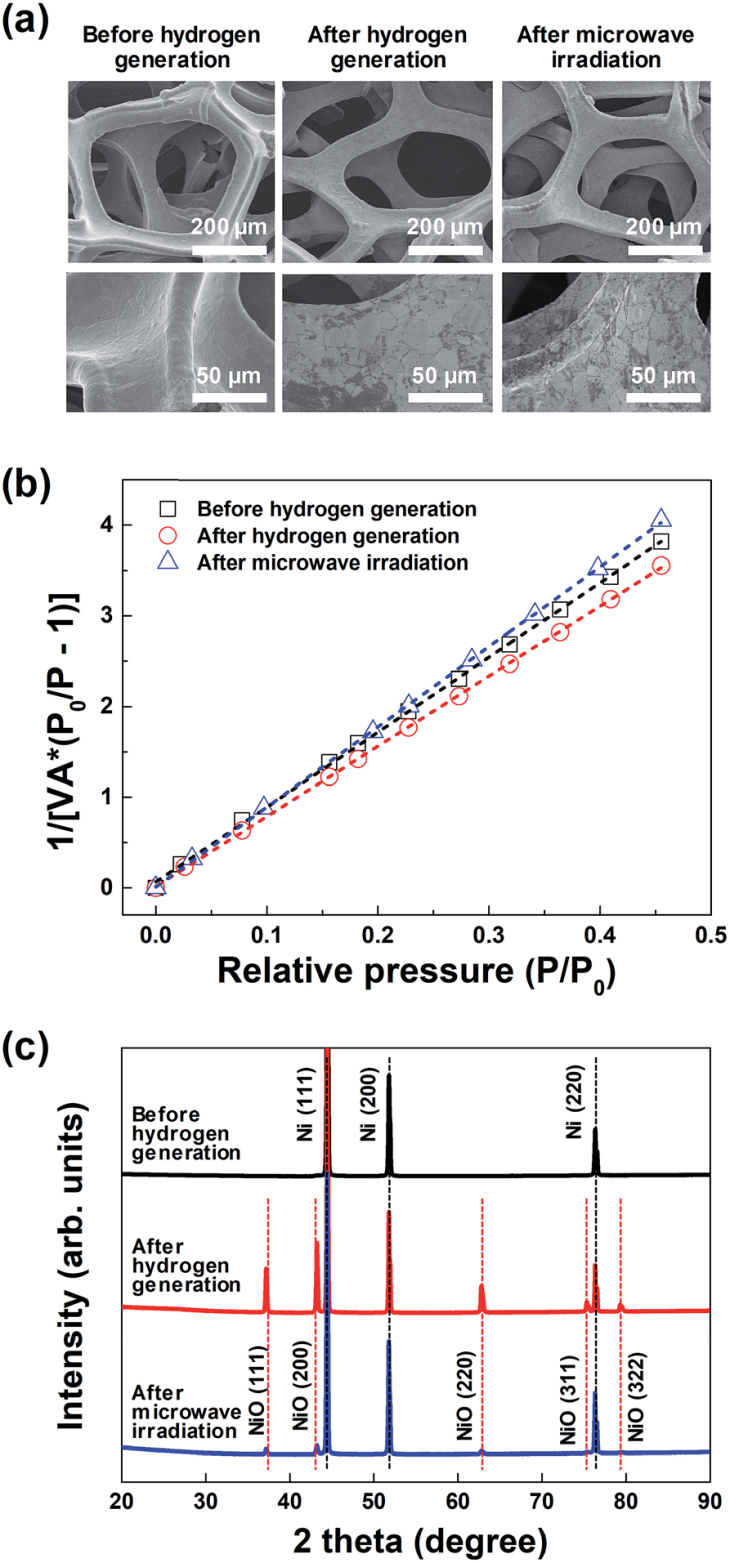
(a) FE-SEM images, (b) BET surface area plots, and (c) XRD spectra of the macroporous nickel-oxide structure before and after hydrogen generation, and after microwave irradiation.

Fig. 3(a) shows the representative FE-SEM images of the macroporous nickel-oxide structure before hydrogen generation, after hydrogen generation, and after microwave irradiation. As shown in the figures, the shape of the 3-D wire-frame-like mesh, with a pore size of ∼230 μm, was maintained before and after hydrogen generation as well as after microwave irradiation. In the enlarged FE-SEM image shown in the bottom in Fig. 3(a), the oxidized surface of the nickel oxide, which showed a smooth surface before hydrogen generation, appears to be a somewhat white-colored area after hydrogen generation. In addition, after microwave irradiation, the reduced white-colored area confirmed that the oxidized surface was slightly reduced. The porous structure of the macroporous nickel-oxide structure was evaluated from the adsorption arms of the isotherms based on the Barrett–Joyner–Halenda (BJH) model. As shown in Fig. 3(b), the measured BET areas of the macroporous nickel-oxide structure before and after hydrogen generation and after microwave irradiation were 0.53, 0.56, and 0.49 m^2^ g^−1^, respectively. From the values of these similar BET areas, it can be deduced that the macroporous nickel-oxide structure maintains the same structure in the two-step thermochemical cycles, oxidation step, and reduction step, indicating that hydrogen gas could be constantly produced during the hydrogen generation/regeneration or reduction processes.

Fig. 3(c) shows the XRD results of the macroporous nickel-oxide structure under three different conditions, before hydrogen generation, after hydrogen generation, and after microwave irradiation (reduction) with PVP. Three main peaks (2*θ*), 44.5°, 51.8°, and 76.1°, corresponding to the (111), (200), and (220) planes of Ni, respectively, were observed on the oxygen-vacancy-rich macroporous nickel-oxide structure before hydrogen generation (ICDD PDF card 87-0712, JCPDS 2001). After hydrogen generation, the peaks corresponding to Ni were slightly reduced and the peaks corresponding to NiO_*x*_ appeared. In other words, five weak peaks (2*θ*), 37.4°, 43.4°, 62.9°, 75.4°, and 79.4°, corresponding to the (111), (200), (220), (311), and (222) crystal planes, respectively, of cubic NiO_*x*_ were observed on the oxygen-rich macroporous nickel-oxide structure after hydrogen generation (JCPDS card no. 73-1523).^21–23^ Microwave irradiation as a reduction process changed the oxygen-rich macroporous nickel-oxide structure to an oxygen-vacancy-rich macroporous nickel-oxide structure. This result can be confirmed from the observation that the XRD peak after microwave irradiation shows that the intensities of the five peaks (2*θ*) corresponding to NiO_*x*_ have decreased. These results indicate that the process of microwave irradiation is effective for releasing abundant oxygen that existed on the macroporous nickel-oxide structure.

**Fig. 4:**
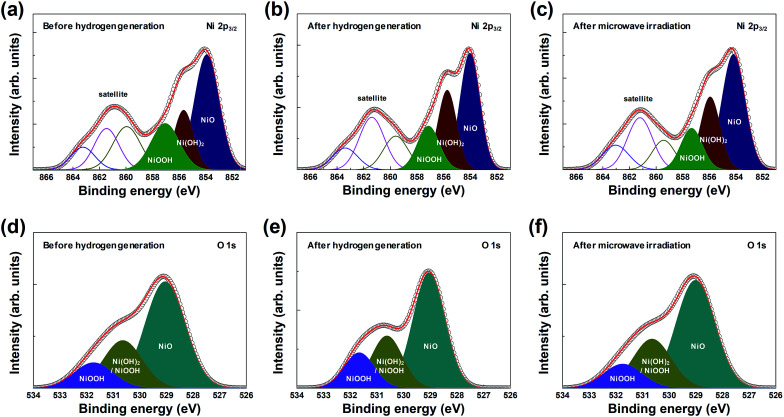
XPS spectra of the macroporous nickel-oxide structure before and after hydrogen generation, and after microwave irradiation in the Ni 2p_3/2_ ((a), (b) and (c), respectively) and O 1s ((d), (e) and (f), respectively) regions.

In addition, the chemical composition and chemical states of the surface of the macroporous nickel-oxide structure were observed by XPS analysis. The macroporous nickel-oxide structure used to generate hydrogen, in this study, reacted with water vapor and formed the oxygen-rich layer on its surface. To continuously regenerate hydrogen, the oxygen-rich layer formed on the nickel surface must be removed again. The oxygen release effect of microwave irradiation was examined by comparing the outermost layer present on the macroporous nickel-oxide structure in three steps, before hydrogen generation, after hydrogen generation, and after microwave irradiation. Fig. 4 shows the XPS spectra of Ni 2p_3/2_ and O 1s before hydrogen generation (Fig. 4(a) and (d)), after hydrogen generation (Fig. 4(b) and (e)), and after microwave irradiation (reduction) with PVP (Fig. 4(c) and (f)). Before hydrogen generation, the pristine macroporous nickel-oxide structure showed a broad satellite peak corresponding to the binding energies of NiOOH (857.1 eV for Ni 2p_3/2_, 530.6 and 531.7 eV for O 1s) in Ni^3+^ and NiO (854.0 eV for Ni 2p_3/2_, 529.1 eV for O 1s) and Ni(OH)_2_ (855.6 eV for Ni 2p_3/2_, 530.6 eV for O 1s) in Ni^2+^.^24–28^ The shake-up-related satellite (broad peak at ∼861 eV for Ni 2p_3/2_) was also observed due to excitation to a higher energy level through the interactions of outgoing electrons and valence electrons.^29,30^ On the contrary, the area ratio of the [Ni(OH)_2_ and NiOOH]/NiO peaks increased to 1.07 after hydrogen generation compared to 0.88 before hydrogen generation because the macroporous nickel-oxide structure that reacted with the water vapor formed an oxygen layer on its surface by absorbing oxygen from the water vapor.^25^ After microwave irradiation, the area ratio of the [Ni(OH)_2_ and NiOOH]/NiO peaks decreased to 0.88. Fig. 4(d)–(f) show the change in area ratios of the [Ni(OH)_2_ and NiOOH]/NiO peaks, with the areas of the NiOOH peak (530.6 and 531.7 eV), the Ni(OH)_2_ peak (530.6 eV), and the NiO peak (529.1 eV) in the O 1s state changing to 0.67 (before hydrogen generation), 0.73 (after hydrogen generation), and 0.67 (after microwave irradiation), respectively. After microwave irradiation with PVP, these three peaks decreased to the level before hydrogen generation due to the reduction effect. Observation of the Ni 2p_3/2_ and O 1s peaks of the macroporous nickel-oxide structure showed whether nickel-oxide was oxygen-saturated or oxygen-deficient. The amount of oxygen vacancies of nickel-oxide reduced after hydrogen generation could be recovered to the state before hydrogen generation by microwave irradiation with PVP. In addition, several redox cycles exhibited chemical compositions similar to the macroporous nickel-oxide structure by microwave irradiation with almost similar chemical composition ratios before hydrogen generation and after microwave irradiation. These results showed that the amount of hydrogen produced remains the same regardless of the number of redox cycles.

**Fig. 5:**
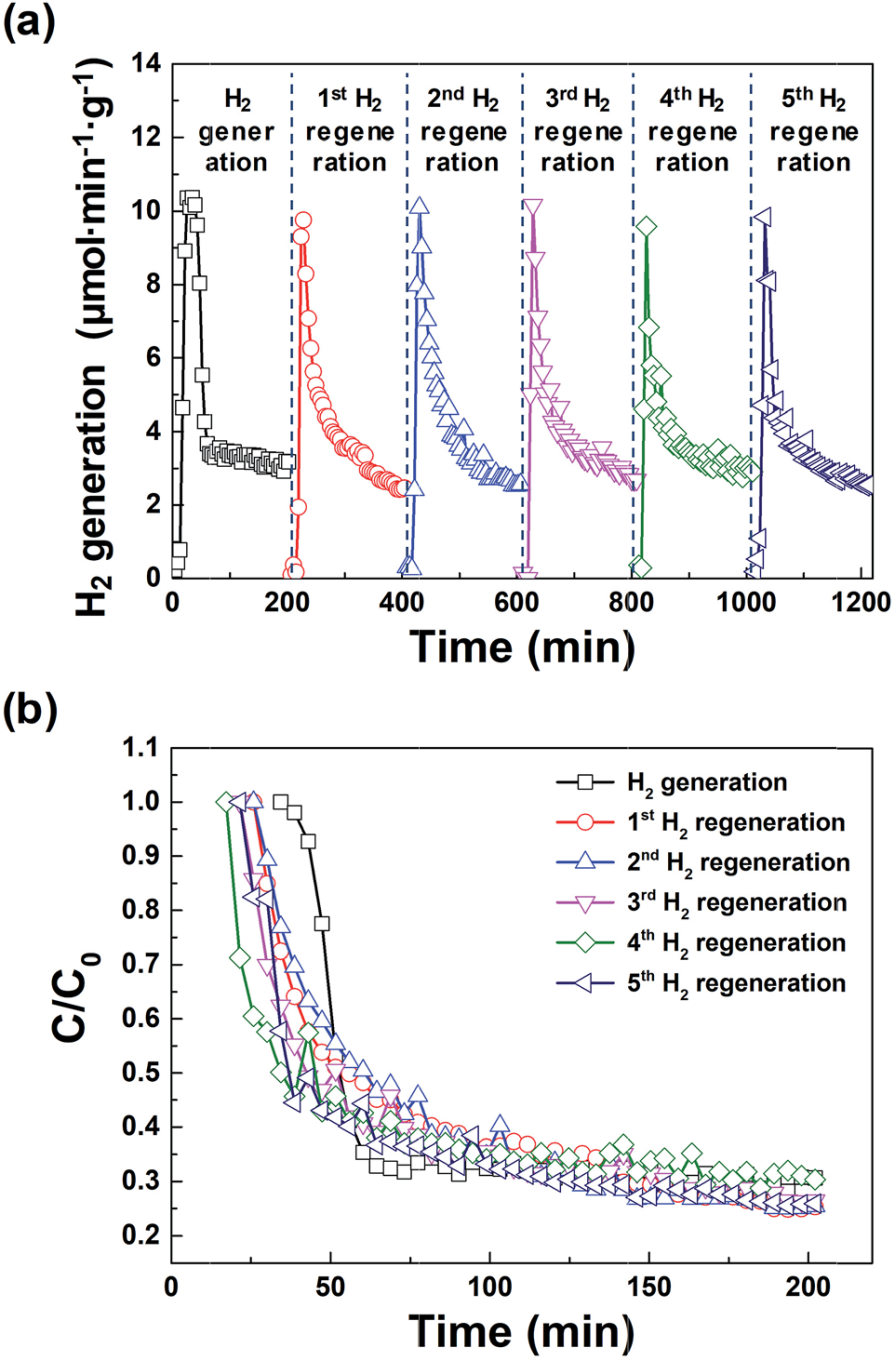
(a) Hydrogen generation characteristics of six repeatable hydrogen generation cycles after the oxygen reduction process under microwave irradiation (regeneration). (b) Time profiles of hydrogen degradation (*C*/*C*_0_) during the six repeatable hydrogen generation cycles.

Fig. 5(a) shows the amount of hydrogen generated from the macroporous nickel-oxide structure during six cycles of hydrogen generation/regeneration, which initially increased sharply, then gradually decreased after 20 min. This is because the surface of the macroporous nickel-oxide structures reacting with the water vapor increasingly oxidizes during hydrogen generation. The amount of hydrogen generated became ∼30% of the initial amount at ∼200 min after the hydrogen generation process; this time was defined as a time point from one cycle to the next cycle. The maximum hydrogen generation rate during the repeated hydrogen generation process was ∼10.0 μmol min^−1^ g^−1^. The total amount of hydrogen produced during one cycle of 200 min was ∼750 μmol g^−1^. Fig. 5(b) shows the degradation rates (*C*/*C*_0_) for hydrogen generation during repetitive hydrogen generation/regeneration cycles by microwave irradiation of the macroporous nickel-oxide structure. It is worth noting that *C*_0_ is the maximum amount of hydrogen and *C* is the amount of hydrogen at a certain time. The similar *C*/*C*_0_ values from the six cycles of hydrogen generation-regeneration proved that this reduction method of microwave irradiation with PVP for the hydrogen regeneration process is very stable.

## 4. Conclusions

In summary, a reduction method for continuous hydrogen regeneration, in which oxygen-saturated metal oxides after hydrogen generation are converted to oxygen-deficient metal oxides again by microwave irradiation, was studied. The macroporous nickel-oxide structure, used as a metal oxide catalyst for hydrogen generation, can produce a maximum hydrogen generation rate of ∼10.0 μmol min^−1^ g^−1^ at 800 °C by the thermochemical water splitting method. After this, it was possible to regenerate hydrogen at almost the same level as the initial hydrogen generation rate under repeated hydrogen generation/regeneration cycles using the reduction process through microwave irradiation with PVP instead of conventional high-temperature heat treatment. Though it will be slightly different depending on the equipment, for instance if temperatures in the range of 1000–2500 °C for 1 h are used for the oxygen reduction process, 7.7–13.9 kJ is required for high-temperature heat treatment. If the proposed microwave irradiation is used with a household microwave oven, the oxygen reduction process can be performed at 0.047 kJ (600 W, 180 s). Thus, microwave irradiation can reduce power consumption by ∼4% compared to high-temperature heat treatment. As a result, through repeated hydrogen regeneration cycles, a total amount of ∼750 μmol g^−1^ of generated hydrogen can be produced per cycle. The amount of generated hydrogen can produce energy 10 times higher than the required energy for microwave irradiation. This has the advantage that the energy consumption required for hydrogen regeneration can be reduced and the hydrogen generation efficiency can be improved.

## Conflicts of interest

There are no conflicts to declare.

## Supplementary Material

## References

[cit1] Muhich C. L., Evanko B. W., Weston K. C., Lichty P., Liang X., Martinek J., Musgrave C. B., Weimer A. W. (2013). Science.

[cit2] Zou Z., Ye J., Sayama K., Arakawa H. (2001). Nature.

[cit3] Stojić D. L., Marčeta M. P., Sovilj S. P., Miljanić Š. S. (2003). J. Power Sources.

[cit4] Ni M., Leung D. Y. C., Leung M. K. H., Sumathy K. (2006). Fuel Process. Technol..

[cit5] Steinfeld A. (2002). Int. J. Hydrogen Energy.

[cit6] Gálvez M. E., Frei A., Albisetti G., Lunardi G., Steinfeld A. (2008). Int. J. Hydrogen Energy.

[cit7] Abanades S., Charvin P., Lemont F., Flamant G. (2008). Int. J. Hydrogen Energy.

[cit8] Roeb M., Sattler C., Klüser R., Monnerie N., de Oliveira L., Konstandopoulos A. G., Agrafiotis C., Zaspalis V. T., Nalbandian L., Steele A., Stobbe P. (2005). J. Sol. Energy Eng..

[cit9] Nakamura T. (1977). Sol. Energy.

[cit10] Xu B., Bhawe Y., Davis M. E. (2012). Proc. Natl. Acad. Sci. U. S. A..

[cit11] Lee S., Hanif Z., Seo K., Lim T., Shin H.-M., Park S., Kim S. H., Kwak S. K., Hong S., Yoon M.-H., Ju S. (2016). J. Mater. Chem. A.

[cit12] Weidenkaff A., Reller A. W., Wokaun A., Steinfeld A. (2000). Thermochim. Acta.

[cit13] Lim T., Ju S. (2017). AIP Adv..

[cit14] Hao Y., Yang C.-K., Haile S. M. (2013). Phys. Chem. Chem. Phys..

[cit15] Chueh W. C., Falter C., Abbott M., Scipio D., Furler P., Haile S. M., Steinfeld A. (2010). Science.

[cit16] Gärtner F., Sundararaju B., Surkus A.-E., Boddien A., Loges B., Junge H., Dixneuf P. H., Beller M. (2009). Angew. Chem., Int. Ed..

[cit17] Gupta M., Paul S., Gupta R. (2009). Acta Chim. Slov..

[cit18] Ryu J., Kim H.-S., Hahn H. T. (2011). J. Electron. Mater..

[cit19] Rao C. N. R., Dey S. (2017). Proc. Natl. Acad. Sci. U. S. A..

[cit20] Le Gal A., Abanades S., Flamant G. (2011). Energy Fuels.

[cit21] Hwang H.-J., Oh K.-H., Kim H.-S. (2016). Sci. Rep..

[cit22] Horikoshi S., Hidaka H., Serpone N. (2001). J. Photochem. Photobiol., A.

[cit23] Lambers E. S., Dykstal C. N., Seo J. M., Rowe J. E., Holloway P. H. (1996). Oxid. Met..

[cit24] Kwon U., Kim B.-G., Nguyen D. C., Park J.-H., Ha N. Y., Kim S.-J., Ko S. H., Lee S., Lee D., Park H. J. (2016). Sci. Rep..

[cit25] Chigane M., Ishikawa M. (1998). J. Chem. Soc., Faraday Trans..

[cit26] Lian K., Thorpe S. J., Kirk D. W. (1992). Electrochim. Acta.

[cit27] Niklasson G. A., Granqvist C. G. (2007). J. Mater. Chem..

[cit28] Li Y., He Y. (2014). RSC Adv..

[cit29] Grosvenor A. P., Biesinger M. C., Smart R. S. C., McIntyre N. S. (2006). Surf. Sci..

[cit30] Biesinger M. C., Payne B. P., Lau L. W. M., Gerson A., Smart R. S. C. (2009). Surf. Interface Anal..

